# Involvement of Cl^−^/HCO_3_^−^ exchanger SLC26A3 and SLC26A6 in preimplantation embryo cleavage

**DOI:** 10.1038/srep28402

**Published:** 2016-06-27

**Authors:** Yong Chao Lu, Jing Yang, Kin Lam Fok, Ying Hui Ye, Liang Jin, Zheng Yun Chen, Xin Mei Zhang, He Feng Huang, Hsiao Chang Chan

**Affiliations:** 1 Department of Obstetrics and Gynecology, Women’s Hospital, Zhejiang University School of Medicine, Hangzhou, Zhejiang, China; 2 The Key Laboratory of Reproductive Genetics, Ministry of Education (Zhejiang University), Hangzhou, China; 3 Department of Assisted Reproduction, Shanghai Ninth People’s Hospital, Shanghai Jiaotong University School of Medicine, Shanghai, China; 4 Epithelial Cell Biology Research Center, School of Biomedical Sciences, Faculty of Medicine, The Chinese University of Hong Kong, Sha Tin, NT, Hong Kong SAR, China; 5 The International Peace Maternity and Child Health Hospital, Shanghai Jiao Tong University School of Medicine, Shanghai, China

## Abstract

Bicarbonate (HCO_3_^−^) is essential for preimplantation embryo development. However, the mechanism underlying the HCO_3_^−^ transport into the embryo remains elusive. In the present study, we examined the possible involvement of Cl^−^/HCO_3_^−^ exchanger in mediating HCO_3_^−^ transport into the embryo. Our results showed that depletion of extracellular Cl^−^, even in the presence of HCO_3_^−^, suppressed embryo cleavage in a concentration-dependent manner. Cleavage-associated HCO_3_^−^-dependent events, including increase of intracellular pH, upregulation of miR-125b and downregulation of p53, also required Cl^−^. We further showed that Cl^−^/HCO_3_^−^ exchanger solute carrier family 26 (SLC26) A3 and A6 were expressed at 2-cell through blastocyst stage. Blocking individual exchanger’s activity by inhibitors or gene knockdown differentially decreased embryo cleavage and inhibited HCO_3_^−^-dependent events, while inhibiting/knocking down both produced an additive effect to an extent similar to that observed when CFTR was inhibited. These results indicate the involvement of SLC26A3 and A6 in transporting HCO_3_^−^ essential for embryo cleavage, possibly working in concert with CFTR through a Cl^−^ recycling pathway. The present study sheds light into our understanding of molecular mechanisms regulating embryo cleavage by the female reproductive tract.

After fertilization, mammalian embryos travel along the oviduct and make their way to the uterus before implantation. During this transit, the pre-implantation embryo undergoes cleavage, an important process producing more blastomere to enable differentiation, blastocyst formation, hatching and implantation^1^,^2^. Thus, preimplantation embryo development is greatly influenced by oviductal and uterine environments. Although evidences have indicated that pregnancy loss is a multi-factorial phenomenon, the biochemical composition of the embryo and maternal environment are the main players to determine pregnancy outcome^3^. While much attention has been paid to understand hormonal influences on embryo development^4^,^5^, much less studies are reported focusing on factors derived from the embryo-maternal environment.

It has been known for decades that female reproductive tract, including the oviduct and uterus, contains high concentrations of HCO_3_^−^ (up to 90 mM), which is much higher than that in most other tissues^6^,^7^,^8^. Impaired HCO_3_^−^ secretion by oviduct epithelium inhibits embryo cleavage and blastocyst formation^9^, indicating an essential role of oviductal and uterine HCO_3_^−^ in embryo development. In preimplantation embryo, HCO_3_^−^ entry has been shown to activate soluble adenylate cyclase in the cytoplasm and triggers in a series of events required for embryo cleavage^9^. However, how HCO_3_^−^ is transported into the embryo remains largely unknown.

Our recent study has demonstrated a crucial role of cystic fibrosis transmembrane conductance regulator (CFTR), an anion channel known to conduct both Cl^−^ and HCO_3_^−^ 10,11, in the process of embryo cleavage and differentiation^1^. Disrupting CFTR function by inhibitors or knockdown decreases intracellular pH (pH_i_), suppresses the sAC/PKA cascade and thus embryo cleavage. CFTR knockout embryo also showed reduced cleavage capacity and blastocyst formation *in vitro* and *in vivo*. These results indicate an important role of CFTR in mediating HCO_3_^−^ transport essential for preimplantation embryo development^1^. However, it remains to be resolved whether CFTR directly conducts HCO_3_^−^ or acts as a Cl^−^ channel working in parallel with a Cl^−^/HCO_3_^−^ exchanger, thereby providing a recycling pathway for the Cl^−^ that is necessary to operate the anion exchanger^12^,^13^,^14^.

The SLC26 gene family encodes for anion transporters that transport a variety of monovalent and divalent anions^15^. Among the 11 members identified, SLC26A3 and SLC26A6 are the two main Cl^−^/HCO_3_^−^ exchangers expressed in various epithelial tissues^16^. Although it has been postulated that Cl^−^/HCO_3_^−^ exchange regulates intracellular pH of embryos^17^, the identity of Cl^−^/HCO_3_^−^ exchanger(s) involved and its exact role in embryo development remain unknown. We undertook the present study to examine the possible involvement of Cl^−^ and Cl^−^/HCO_3_^−^ exchangers, particularly the two SLC26A family members, in preimplantation embryo development.

## Results

### Embryo cleavage requires both Cl^−^ and HCO_3_^−^

**Figure 1:**
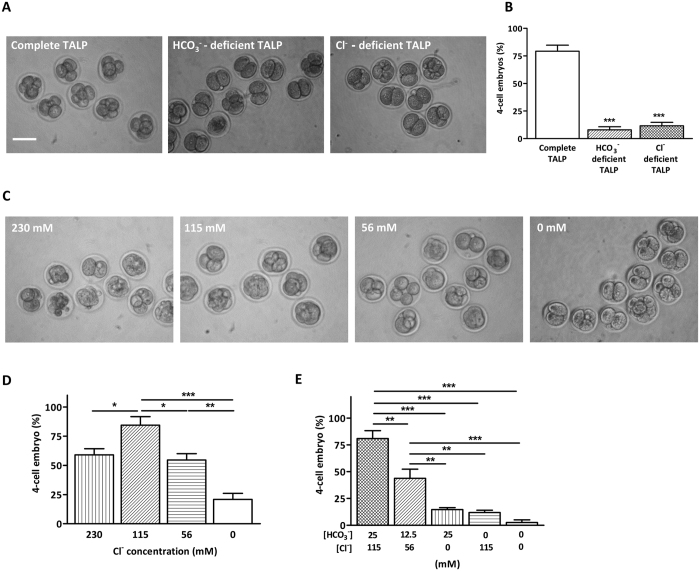
Cl^−^ and HCO_3_^−^- are both required for embryo cleavage. (**A**) Four-cell embryo formation after 12 hours of 2-cell embryo culture in complete, Cl^−^ deficient or HCO_3_^−^ deficient TALP medium (complete TALP: 25/32 embryos; HCO_3_^−^ deficient TALP: 4/43 embryos; Cl^−^ deficient TALP: 7/39 embryos). Scale bar: 100 μm. (**B**) Summary of the results from A. *** indicates *P* < 0.001 (by one-way ANOVA, *n* = 4) when compared with control. (**C**) Four-cell embryo formation after 12 hours of 2-cell embryo culture in TALP medium with varying concentrations of Cl^−^ and constant HCO_3_^−^ concentration (25 mM) (230 mM: 20/35 embryos; 115 mM: 29/33 embryos; 56 mM: 20/37 embryos; 0 mM: 7/30 embryos). Scale bar: 100 μm. (**D**) Summary of the results from C. * indicates *P* < 0.05, ** indicates *P* < 0.01, *** indicates *P* < 0.001 (by one-way ANOVA, *n* = 4). (**E**) Summary of the synergistic effect of HCO_3_^−^ and Cl^−^ on embryo cleavage (0 mM HCO_3_^−^ and 0 mM Cl^−^: 1/40 embryos; 12.5 mM HCO_3_^−^ and 56 mM Cl^−^: 18/41 embryos; 0 mM HCO_3_^−^ and 115 mM Cl^−^: 5/42 embryos; 25 mM HCO_3_^−^ and 0 mM Cl^−^: 6/41 embryos; 25 mM HCO_3_^−^ and 115 mM Cl^−^: 30/37 embryos). ** indicates *P* < 0.01, *** indicates *P* < 0.001 (by one-way ANOVA, *n* = 4).

To test the possible involvement of Cl^−^/HCO_3_^−^ exchangers, we first examined the requirement of Cl^−^ in cleavage and blastocyst formation. When 2-cell mouse embryos were incubated in Cl^−^ deficient TALP for 12 h, there was a significant decrease in the percentage of 4-cell embryo compared to that in the control with complete TALP (Fig. 1A,B). The inhibiting effect was similar to that observed in HCO_3_^−^-deficient TALP medium (Fig. 1A,B). Next, we examined the effect of varying Cl^−^ concentrations while maintaining constant HCO_3_^−^ concentration, or *vice versa*, on embryo cleavage. In the first set of experiment, TALP medium with different HCO_3_^−^ concentrations and a constant Cl^−^ concentration (115 mM) was used. Consistent with previous study, embryo cleavage was reduced as the concentration of HCO_3_^−^ in the TALP decreased, with less than 10% embryos develop into 4-cell stage when HCO_3_^−^-deficient TALP was used (Supplemental Fig. S1), confirming an essential role of HCO_3_^−^ in the process of cleavage. The second set of experiments was performed using TALP medium with varying Cl^−^ concentrations and constant HCO_3_^−^ concentration (25 mM). The results showed that embryo cleavage decreased with decreasing Cl^−^ concentrations in the medium, despite the presence of sufficient HCO_3_^−^ (Fig. 1C,D). These results indicate that embryo cleavage requires both Cl^−^ and HCO_3_^−^. To further discern whether the Cl^−^ and HCO_3_^−^ act synergistically or dependently on embryo cleavage, we examined the percentage of embryo cleavage in medium containing half effective concentration of both HCO_3_^−^ (12.5 mM) and Cl^−^ (56 mM) (Fig. 1E). The result showed that the percentage of embryo cleavage in medium containing half effective concentration of both ions was similar to that containing half effective concentration of either Cl^−^ (Fig. 1D) or HCO_3_^−^ ion (Supplementary Fig. S1), suggesting that the function of Cl^−^ on embryo cleavage were largely, if not all, dependent on HCO_3_^−^.

### Effect of Cl^−^ and HCO_3_^−^ on increased pH_i_ during embryo cleavage

**Figure 2:**
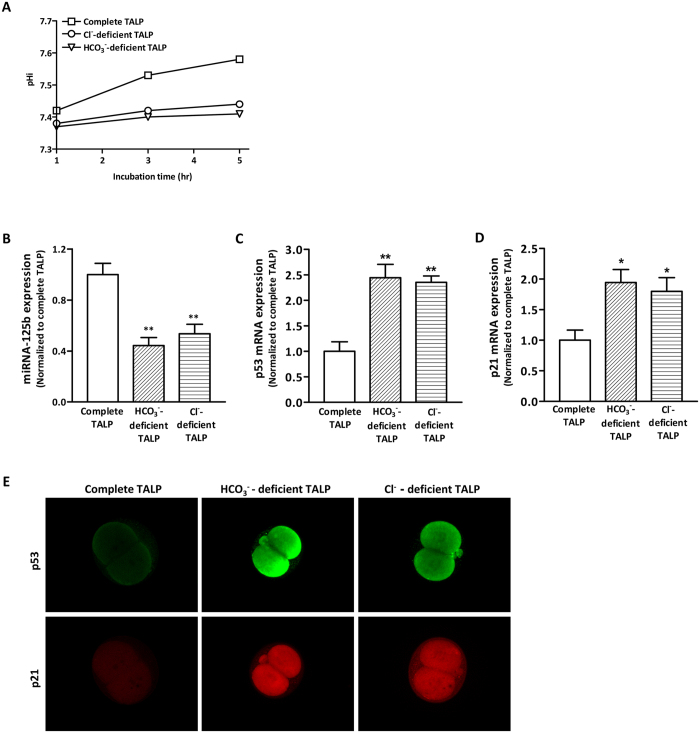
Involvement of Cl^−^ in HCO_3_^−^-dependent signaling essential for embryo cleavage. (**A**) Intracellular pH measurement of 2-cell embryos incubated in Cl^−^-deficient, HCO_3_^−^-deficient or complete TALP medium. Five micromolar BCECF was loaded for 30 min before determination of the pH_i_. The time course changes in pH_i_ were shown (10 embryos/group). The experiments were repeated 4 times. (**B–D**) Quantitative real-time PCR result showing the expression of miRNA-125b (**B**), p53 (**C**) and p21 (**D**) in embryos incubated in HCO_3_^−^-free or Cl^−^-free condition compared to that incubated in complete TALP medium. ** indicates *P* < 0.01 (by one-way ANOVA, ~100 embryos in each experiment, *n* = 4). (**E)** Representative immunofluorescence results showing increased expression of p53 (green) and p21 (red) in 0 mM HCO_3_^−^ condition or 0 mM Cl^−^ condition (5–10 embryos/group). Scale bar, 150 μm.

Next, we examined if Cl^−^ is required for the transport of HCO_3_^−^ into the embryo. The entry of HCO_3_^−^ has been reported to lead to an increase in pH_i_ of blastomere of 2-cell embryo^18^,^19^, an early hallmark event in the process of embryo cleavage. Therefore, we used pH_i_ as an indicator of HCO_3_^−^ entry. Indeed, as shown in Fig. 2A, the pH_i_ increased with time when 2-cell embryos were incubated in complete TALP, while remained almost unchanged in those incubated in HCO_3_^−^-deficient TALP. Interstingly, pH_i_ also remained unchanged when the embryos were incubated in Cl^−^-deficient TALP, suggesting that Cl^−^ deficiency impairs HCO_3_^−^ entry into the embryos.

### Effect of Cl^−^ on cleavage-related gene expression

In preimplantation embryos, HCO_3_^−^ influx has been shown to act as an environmental cue in activating the expression of miR125b, an effector of HCO_3_^−^-dependent signaling essential for embryo development^1^. High level of miR125b expression suppresses the expression of p53, the latency of which is required for normal embryo development^20^. To assess the involvement of Cl^−^ in mediating these HCO_3_^−^-dependent gene expressions and embryo development, 2-cell embryos were separately incubated in Cl^−^/HCO_3_^−^ complete TALP, HCO_3_^−^-deficient TALP or in Cl^−^-deficient TALP for 6 h and the gene expression was analysed by realtime PCR. The results showed that embryos incubated in Cl^−^/HCO_3_^−^ complete TALP were accompanied by a relatively high level of miR-125b expression, consistent with that normally observed during cleavage^1^. However, embryos incubated in HCO_3_^−^-deficient or Cl^−^-deficient TALP showed significantly decrease in miR-125b expression (Fig. 2B) with elevation of its target p53^21^ (Fig. 2C). The expression of p21, a downstream target of p53^1^, was also significantly increased (Fig. 2D). Consistent with the realtime PCR results, p53 and p21 proteins were markedly increased in embryos cultured under HCO_3_^−^ -deficient or Cl^−^-deficient TALP (Fig. 2E). These results suggest that Cl^−^ regulates HCO_3_^−^-dependent events essential for embryo development.

### Expression and localization of SLC26A3 and SLC26A6 in preimplantation embryo

**Figure 3:**
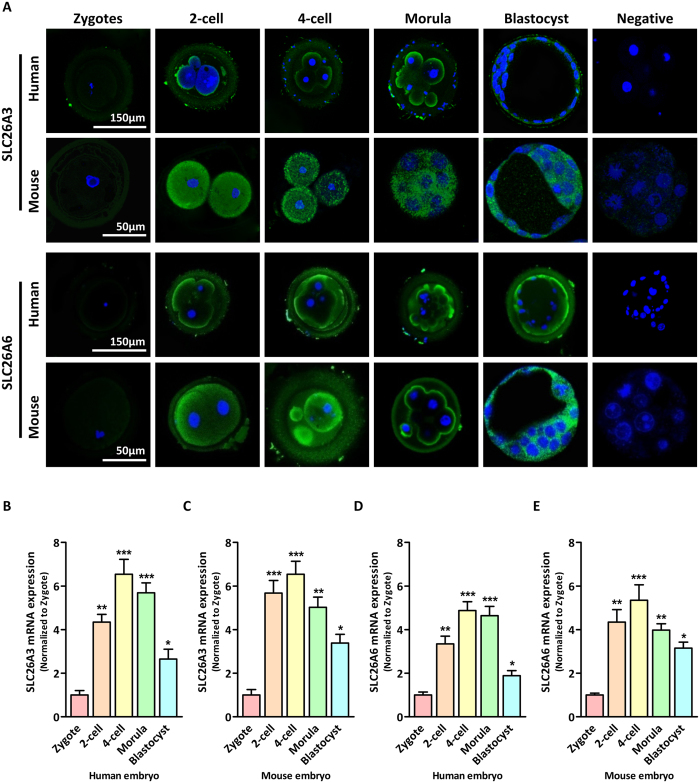
Expression of SLC26A3 and SLC26A6 in preimplantation embryos. (**A**) Immunofluorescent results showing the localization of SLC26A3 (upper panel, green) and SLC26A6 (lower panel, green) protein in different stages of human and mouse preimplantation embryos. Nuclei were counterstained with DAPI (blue). (**B–E**) Quantitative real-time PCR results showing the levels of SLC26A3 (**B**,**D**) and SLC26A6 (**C**,**E**) mRNA expression during human (**B,C**) and mouse (**D,E**) preimplantation embryo development. * indicates *P* < 0.05, ** indicates *P* < 0.01, *** indicates *P* < 0.001 (by one-way ANOVA, ~100 embryos in each experiment, *n* = 4).

We have previously demonstrated an important role of CFTR in transporting HCO_3_^−^ necessary for embryo cleavage^1^. The observed Cl^−^-dependent cleavage and its regulation of HCO_3_^−^-dependent cellular alkalinzation and gene expressions suggest the involvement of Cl^−^/HCO_3_^−^exchanger(s) working in concert with CFTR in transporting HCO_3_^−^. SLC26 mutations have been associated with subfertility in human^22^ and a SLC26A member had been shown to work in concert with CFTR in sperm^23^. Therefore, we screened the expression of SLC family members in preimplantation embryo. The results showed that the expression of SLC26A3 and SLC26A6 mRNA were significantly higher than other SLC members, which were reported to be expressed in early stage embryo^24^ (Supplemental Fig. S2). Thus, we focused on SLC26A3 and SLC26A6 and examined their potential role in embryo development. We characterized the expression of SLC26A3 and SLC26A6 by indirect immunofluorescence staining and realtime PCR in different stages of embryo. As shown in Fig. 3A, SLC26A3 and SLC26A6 protein expression was hardly detected in zygote stage but markedly increased at 2-cell stage and continued to express at blastocyst stage in both human and mouse preimplantation embryos. Consistent with the immunofluorescence staining, both SLC26A3 and SLC26A6 mRNA showed a marked increase in 2-cell embryo compared to the zygote stage. Their expression continues to increase until 4-cell stage and tailed off at blastocyst stage (Fig. 3B–E). The observed temporal changes in the expression of SLC26A3 and SLC26A6 in the early stages of embryo cleavage suggest their potential involvements in embryo cleavage.

### Effects of inhibitors of SLC26A3 and SLC26A6 on HCO_3_^−^-dependent embryo cleavage

**Figure 4:**
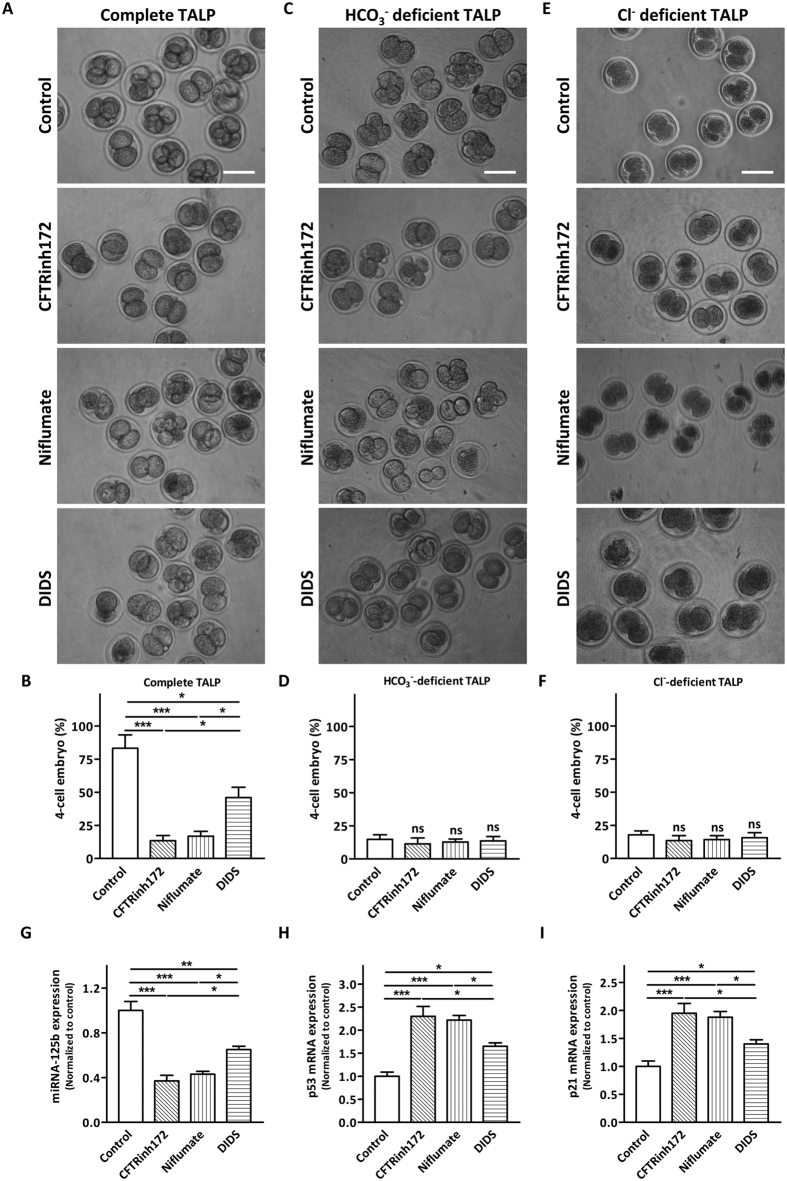
Effects of SLC26A3 and SLC26A6 inhibitors on embryo cleavage and HCO_3_^−^-dependent signalling cascade. (**A–F**) Effects of inhibiting CFTR (CFTRinh172, 10 μM), SLC26A3 (niflumate, 20 μM) and SLC26A6 (DIDS, 20 μM) on preimplantation embryo cleavage in complete TALP (**A,B**) control: 32/38 embryos; CFTRinh172: 6/34 embryos; niflumate: 9/35 embryos; DIDS: 14/40 embryos), HCO_3_^−^-deficient TALP (**C,D**) control: 5/30 embryos; CFTRinh172: 7/31 embryos; niflumate: 4/32 embryos; DIDS: 6/31 embryos) and Cl^−^-deficient TALP (**E,F**, control: 6/32 embryos; CFTRinh172: 4/31 embryos; niflumate: 5/30 embryos; DIDS: 6/33 embryos). Scale bar: 100 μm. Summary of the results are shown on the right panel (**B**,**D**,**F**). ns indicates *P* > 0.05, * indicates *P* < 0.05, ** indicates *P* < 0.01, *** indicates *P* < 0.001 (by one-way ANOVA, *n* = 4). (**G–I**) Quantitative real-time PCR showing the expression of miRNA-125b (**G**), p53 (**H**) and p21 (**I**) after CFTRinh172, niflumate or DIDS treatment. * indicates *P* < 0.05, ** indicates *P* < 0.01 (by one-way ANOVA, ~100 embryos in each experiment, *n* = 4).

To test the possible involvement of SLC26A3 and SLC26A6 in embryo cleavage, 2-cell embryos were treated with niflumate (inhibitor of SLC26A3) or DIDS (inhibitor of SLC26A6) for 12 h and the formation of 4-cell embryo was counted. As shown in Fig. 4A,B, 20 μM of niflumate or DIDS treatment significantly decreased the percentage of 4-cell embryo formed compared to the control. The inhibiting effects of niflumate or DIDS were dose-dependent (Supplementary Fig. S3). The degree of inhibition was comparable in niflumate-treated group and CFTR inhibitor-treated group, which was significantly greater than that of the DIDS-treated group. Of note, the inhibiting effects of niflumate, DIDS and CFTR inhibitor were not observed when embryos were incubated in HCO_3_^−^-deficient TALP (Fig. 4C,D) or in Cl^−^-deficient TALP (Fig. 4E,F), suggesting that the effect of these inhibitors were attributed to the blockage of Cl^−^ and HCO_3_^−^ transport. Further, blocking SLC26A3 and SLC26A6 suppressed the expression of miR-125b (Fig. 4G) and elevated the expression of p53 and p21 (Fig. 4H,I). The effect of blocking SLC26A3 on HCO_3_^−^-dependent gene expression was similar to that of blocking CFTR (Fig. 4G–I). These results suggest possible involvement of SLC26A3 and SLC26A6 working in concert with CFTR in regulating HCO_3_^−^-dependent embryo cleavage.

### Effects of SLC26A3 and SLC26A6 knockdown on embryo cleavage

**Figure 5:**
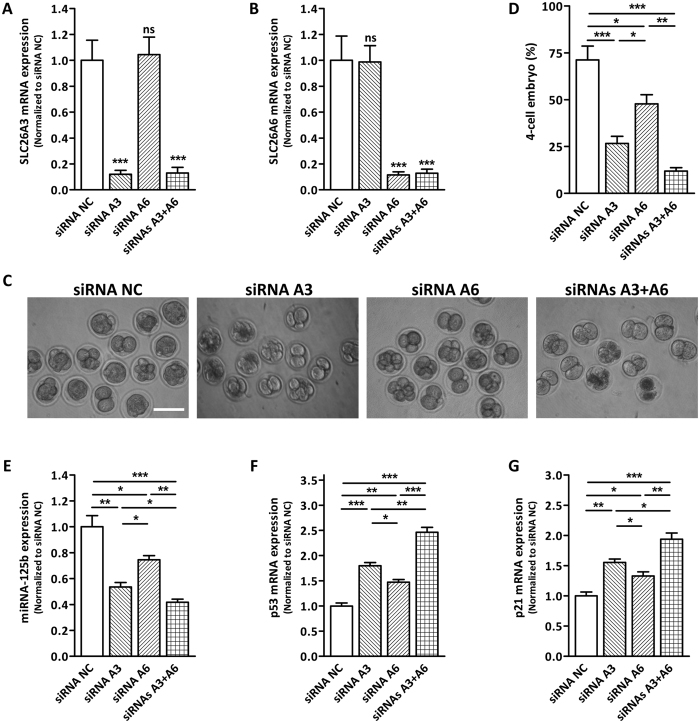
Effect of SLC26A3 and/or SLC26A6 knockdown on embryo cleavage and HCO_3_^−^-dependent signalling cascade. **(A,B**) Quantitative real-time PCR result showing the expression of SLC26A3 (**A**) and SLC26A6 (**B**) mRNA in embryos after control siRNA (siRNA NC), SLC26A3 siRNA, SLC26A6 siRNA or both SLC26A3 and SLC26A6 siRNA injection at the 2-cell stage. *** indicates *P* < 0.001 (by one-way ANOVA, *n* = 4). (**C**) Four-cell embryo formation after 12 hours of embryo culture following siRNA NC, SLC26A3 siRNA, SLC26A6 siRNA or both SLC26A3 and SLC26A6 siRNA injection at 2-cell stage (siRNA NC: 32/45 embryos; SLC26A3 siRNA: 11/44 embryos; SLC26A6 siRNA: 21/40 embryos; SLC26A3 + A6 siRNA: 5/42 embryos). Scale bar: 100 μm. (**D**) Summary of the results from C. * indicates *P* < 0.05, ** indicates *P* < 0.01, *** indicates *P* < 0.001 (by one-way ANOVA, *n* = 4). (**E–G**) Quantitative real-time PCR showing the expression of miRNA-125b (**E**), p53 (**F**) and p21 (**G**) after siRNA NC, SLC26A6 siRNA, SLC26A3 siRNA or both SLC26A3 and SLC26A6 siRNA injection. * indicates *P* < 0.05, ** indicates *P* < 0.01, *** indicates *P* < 0.001 (by one-way ANOVA, ~100 embryos in each experiment, *n* = 4).

We further knocked down SLC26A3 and/or SLC26A6 by microinjecting siRNAs into blastomere of 2-cell embryo to confirm their effect on embryo cleavage. Quantitative real-time PCR results showed that SLC26A3 and SLC26A6 could be effectively knockdown individually or simultaneously by injecting siRNA targeting one subtype or co-injecting two siRNAs (Fig. 5A,B). As shown in Fig. 5C,D, SLC26A3 or SLC26A6 knockdown both markedly reduced the formation of 4-cell embryos with SLC26A3 knockdown showing a significantly more potent effect compared to that of SLC26A6 knockdown. Double knockdown of SLC26A3 and SLC26A6 further inhibited the formation of 4-cell embryos compared to the individual knockdown group, suggesting that SLC26A3 and SLC26A6 might each contribute to embryo cleavage. Consistent with the phenotypic data, individual knockdown of SLC26A3 or SLC26A6 suppressed miR-125b expression and increased p53 and p21 mRNA expression, while double knockdown showed an additive effect (Fig. 5E–G). These results indicate that both SLC26A3 and SLC26A6 play important, yet independent role during embryo cleavage.

## Discussion

**Figure 6:**
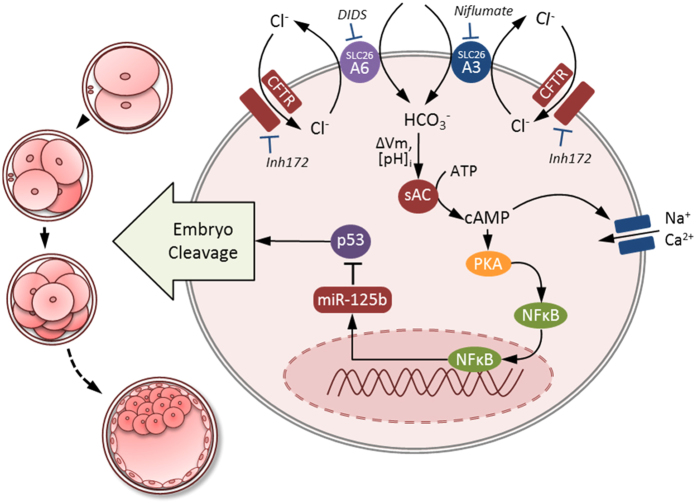
SLC26A3 and SLC26A6 work in concert with CFTR in regulating HCO_3_^−^ transport in preimplantation embryo. Working model for the regulation of early embryo cleavage by SLC26A3 and SLC26A6 in CFTR/HCO_3_^−^-dependent activation of miR-125b. The HCO_3_^−^ influx is mediated by SLC26A3 and SLC26A6 with an exchange of 2Cl^−^/ HCO_3_^−^. Apart from its reported role in conducting HCO_3_^−^ directly, CFTR act as a Cl^−^ channel to provide a recycling pathway for Cl^−^ that is required for SLC26A3 and SLC26A6 function. The sites of action for inhibitors CFTRinh172, niflumate, and DIDS, as well as the intracellular HCO_3_^−^-dependent events, are also shown. HCO_3_^−^ influx mediated by the cooperative action of SLC26A3, SLC26A6 and CFTR activates soluble adenylyl cyclase (sAC) which increase the level of cAMP. This in turn activates PKA/NFkB signaling cascade which increases the expression of miR125b. Expression of miR125b is required for embryo cleavage through suppressing the expression of p53 and p21. Vm, membrane potential; [pH]i, intracellular pH.

Although it is established that HCO_3_^−^ plays an essential role in embryo development before implantation^1^,^9^,^25^,^26^, the present study has demonstrated an equally important role of Cl^−^ in the process by mediating the entry of HCO_3_^−^ that is required for embryo cleavage. To initiate cleavage, HCO_3_^−^ has to first enter into blastomere of embryo and act on an HCO_3_^−^ sensor, sAC, which in turn triggers the PKA/NFkB signaling cascade. Activation of NFkB elevates the expression of miR-125b, which suppresses the expression of p53 and p21, leading to embryo cleavage^1^. In the present study, we showed that some of the key events in this cascade such as increased pH_i_, activation of miR-125b and downregulation of p53, which have been previously shown to be dependent on HCO_3_^−^, were also Cl^−^-dependent. The dependence of the cleavage-associated events on Cl^−^, in addition to HCO_3_^−^, suggests the involvement of Cl^−^/HCO_3_^−^ exchangers in mediating the entry of HCO_3_^−^. Using pharmacological and genetic approaches, we showed that SLC26A3 and SLC26A6 were two Cl^−^/HCO_3_^−^ exchangers required for HCO_3_^−^ entry and HCO_3_^−^-dependent signalling cascades. The operation of these Cl^−^/HCO_3_^−^ exchangers requires CFTR acting as a Cl^−^ channel to provide a recycling pathway for Cl^−^. Defects in either the Cl^−^/HCO_3_^−^ exchange or the CFTR-mediated Cl^−^ recycling pathway would impair HCO_3_^−^ entry, disrupt HCO_3_^−^-dependent signalling cascade and suppress embryo cleavage (Fig. 6).

Despite their similar role as Cl^−^/HCO_3_^−^ exchanger, the stoichiometry of Cl^−^/HCO_3_^−^ exchange by SLC26A3 and SLC26A6 is different. It has been shown that SLC26A3 mediates 2 Cl^−^/1 HCO_3_^−^ exchange while SLC26A6 mediates 1 Cl^−^/2 HCO_3_^−^ exchange^27^. Interestingly, despite similar knockdown efficiency, knockdown of SLC26A3 has a more potent inhibiting effect on embryo cleavage compared to SLC26A6 knockdown (Fig. 5), suggesting a more critical role of SLC26A3 in embryo cleavage. Moreover, double knockdown of SLC26A3 and SLC26A6 showed an additive effect compared to individual knockdown. These results suggest that both SLC26A3 and SLC26A6 may each contribute to HCO_3_^−^ entry required for embryo cleavage while the contribution from SLC26A3 appears to be predominant. The reason behind the preference for a slow kinetic HCO_3_^−^ entry path through SLC26A3 may be accounted for the observed gradual increase in the intracellular alkalinzation in hours in preimplantation embryos (Fig. 2A). SLC26A6 may play a minor or alternative role, which requires further investigation.

The presently demonstrated role of SLC26A3 and SLC26A6 in mediating HCO_3_^−^ entry required for embryo cleavage may have implications in fertility diagnosis or prediction of pregnancy outcome. It has been demonstrated that patients carrying mutations in SLC26A3 gene suffered congenital chloride diarrhea^28^. SLC26A3 knockout mice also exhibit subfertility, however, whether SLC26A3 is associated with other forms of defects in fertility other than male infertility remains unexplored. Our results have clearly shown an important role of SLC26A3 in embryo cleavage and thus preimplantation embryo development. Therefore, we propose that SLC26A3 could be a potential biomarker for predicting pregnancy failure associated with embryo development blockage.

In conclusion, the present results have demonstrated the importance of a maternal environmental factor, Cl^−^, in addition to the previously demonstrated HCO_3_^−^, in preimplantation embryo development. Cl^−^ is required for the operation of Cl^−^/HCO_3_^−^ exchangers, SLC26A3 and/or SLC26A6, for the transport of HCO_3_^−^ into embryos required for the activation of miR-125 and subsequent inactivation of p53/p21 during embryo cleavage. Therefore, in addition to the previously demonstrated important role of CFTR, these anion exchangers, particularly SLC26A3, may play equally important role in regulating HCO_3_^−^ entry and thus embryo cleavage, defect or mutation of which may lead to pregnancy loss due to disrupted embryo development.

## Materials and Methods

### Human embryo samples

Human preimplantation embryos were donated by patients who had a successful pregnancy from an *in vitro* fertilization program at Women’s Hospital, School of Medicine, Zhejiang University. Donation was voluntary and informed consent was given. All human-related procedures were carried out in accordance with guidelines approved by the Ethics Committee for Research on Human Subjects of Zhejiang University.

### Animals

All animal-related procedures were carried out in accordance with the Institutional Guide for Laboratory Animals established by the Animal Care and Use Committee (ACUC), and were approved by the ACUC of the School of Medicine, Zhejiang University (Approval number: ZJU2015-415-05). The mice were housed under a 12/12-h light/dark cycle at 25 ± 0.5 °C and 50–60% humidity, and were fed ad libitum with a standard diet and water.

### Embryo recovery and culture

Female ICR mice (8-week-old) were superovulated by intraperitoneal injections of 10 IU pregnant mares’ serum gonadotrophin (PMSG, Hangzhou Animal Pharmaceutical Factory, Hangzhou, Zhejiang, China), followed by 10 IU human chorionic gonadotrophin (hCG, Hangzhou Animal Pharmaceutical Factory) at 48 h after PMSG injection. Females were caged with ICR males (10-week-old) immediately following hCG injection. Embryos at different stages were obtained by sacrificing the mice at indicated time points after hCG injection; zygote-18 h, 2-cell-44 h, 4-cell-56 h, morula-80 h, blastocyst-92 h. Blastocysts were collected by flushing the uterus with TALP-HEPES medium; embryos at other stages were collected by flushing the oviducts with TALP-HEPES from the infundibular end. The embryos were transferred to TALP medium and cultured under 5% CO_2_ at 37 °C. Unless otherwise specified, all embryos were cultured in the presence of 25 mM HCO_3_^−^ and 115 mM Cl^−^. For development assessment, 2-cell embryos were cultured for 12 h untill 4-cell stage and numbers of blastomere were counted and recorded at that time. For analysis of miR-125b, p53, p21, embryos were collected after various treatments for 6 h.

### Measurement of pH_i_ in embryos

The level of pH_i_ was determined at 2-cell embryos stage by loading with 2′, 7-bis-2 (carbosyethyl)-5-(and-b)-carboxyfluorescence, acetoxymethyl ester (BCECF, B8806, Sigma-Aldrich, St. Louis, MO, USA) as previously described (Xu *et al.*, 2007), with minor modifications. Briefly, 2-cell embryos were obtained and were cultured in complete TALP, Cl^−^-deficient TALP and in HCO_3_^−^-deficient TALP in 5% CO_2_ incubator at 37 °C. When needed, 5 μM BCECF was added in the medium and incubated with embryos for an additional 30 min. Following this, the embryos were washed twice to remove free dye before pH_i_ measurement. Fluorescence was detected by an excitation ratio of 490:440 nm (emission, 520 nm) using a fluorescence microscope photometry systems (FMPS, Nikon, Tokyo, Japan). Calibration was performed according to the method previously used (Fraire-Zamora and Gonzalez-Martinez, 2004), with modification. Briefly, at the end of each trace, 10 μl of 5 mM nigericin was added to permeabilize the blastomeres, the pH was then acidified with 10 μl of HCl, and the measured pH values (determined with a conventional pH meter) were compared with the corresponding ratio values. Usually, we make three additions before ending the experiment. These data were analyzed using Prism 5.0 software (GraphPad Software, San Diego, CA) to convert ratios to pH values.

### SLC26A3/A6 siRNA microinjection to mouse 2-cell embryos

The siRNA negative control (siRNA NC, sc-37007, Santa Cruz Biotechnology, CA, USA), SLC26A3 siRNA (sc-45544, Santa Cruz Biotechnology) or SLC26A6 siRNA (sc-108024, Santa Cruz Biotechnology) was dissolved in RNase-free water. Each blastomere of two-cell embryos was injected with 50 fmol of siRNA solution at the two-cell stage using the micromanipulation system. Formation rate of 4-cell embryo was accessed 12 h after injection.

### Quantitative real-time PCR

Total RNA extraction and reverse transcription (RT) from 100 embryos for each group were performed using Cells-to-cDNA^TM^ II Kit as described (Ambion, Austin, TX, USA). Quantitative real-time PCR was performed by using the ABI Prism 7900HT (Applied Biosystems, Carlsbad, CA, USA). The specific primers were provided by Sangon, Shanghai, China. The full list of primer sequences (mouse p53, p21, SLC26A3, SLC26A6 and GAPDH; human SLC26A3, SLC26A6 and GAPDH) are shown in Supplemental Table 1. Quantitative real-time PCR for miR-125b was carried out with microRNA assay kit (Assay ID: 000449, Applied Biosystems, Carlsbad, CA, USA). snoRNA202 was used as miRNA control (Assay ID: 001232, Applied Biosystems, Carlsbad, CA, USA).

### Immunofluorescence staining and confocal microscopy

The embryos were fixed with 4% paraformaldehyde for 1 h and then permeabilized with 0.1% Triton X-100 in phosphate-buffered saline (PBS) for 30 min. After incubation with 10% goat serum for 1 h to block the nonspecific antigen, the embryos were incubated with primary antibodies: polyclonal goat anti-SLC26A3 primary antibody (1:200 dilution) (sc-34939, Santa Cruz Biotechnology) or goat anti-SLC26A6 primary antibody (1:200 dilution) (sc-26728, Santa Cruz Biotechnology) or goat anti-p53 polyclonal antibody (sc-1312, 1:100, Santa Cruz Biotechnology, Santa Cruz, CA, USA) or rabbit anti-p21 polyclonal antibody (sc-397, 1:200, Santa Cruz Biotechnology) at 4 °C overnight. After incubation, the embryos were washed with PBS, and then incubated with secondary antibodies: FITC-conjugated donkey anti-goat antibody (1:500, sc-3853, Santa Cruz Biotechnology) or Alexa 488 rabbit anti-goat IgG (A27012, 1:500, invitrogen, Rockford, IL, USA) or Alexa 568 goat anti-rabbit IgG (A11011, 1:500, invitrogen) at room temperature for 45 min, followed by nuclear staining with 4′, 6-diamidino-2-phenylindole (DAPI, 1:1000, D9542, Sigma-Aldrich) for 20 min. For the negative controls, we incubated the embryos in PBS without the addition of the primary antibody to determine the levels of non-specific fluorescence. Finally, the fluorescence images were analysed using a Zeiss LSM 510 META laser scanning confocal microscope (Carl Zeiss, Thornwood, NY, USA).

### Statistical analysis

Each experiment was repeated at least three times. Results were presented as means ± standard error (SEM). Student’s unpaired *t*-test was used for two-group comparison. One-way analysis of variance (ANOVA) followed by Tukey’s *post-hoc* test was used when comparing three or more groups. A probability of *P* < 0.05 was considered to be statistically significant.

## Additional Information

**How to cite this article**: Lu, Y. C. *et al.* Involvement of Cl^−^/HCO_3_^−^ exchanger SLC26A3 and SLC26A6 in preimplantation embryo cleavage. *Sci. Rep.* **6**, 28402; doi: 10.1038/srep28402 (2016).

## Supplementary Material

Supplementary Information
