# Supplementary material for: Involvement of Cl−/HCO3− exchanger SLC26A3 and SLC26A6 in preimplantation embryo cleavage
Source: Sci Rep. 2016 Jun 27;6:28402. doi: 10.1038/srep28402 (PMC4921817; doi:10.1038/srep28402)
Supplement: Supplementary Information [file srep28402-s1.pdf]

Involvement of Cl^-/HCO_3^- exchanger SLC26A3 and SLC26A6 in preimplantation embryo cleavage

Yong Chao Lu[1,5,†], Jing Yang[3†], Kin Lam Fok[2], Ying Hui Ye[1,5], Liang Jin[1,5], Zheng Yun Chen[1], Xin Mei Zhang[1], He Feng Huang[4,5*], Hsiao Chang Chan[2,*]

[1]Department of Obstetrics and Gynecology, Women's Hospital, Zhejiang University School of Medicine, Hangzhou, Zhejiang, China

[2]Epithelial Cell Biology Research Center, School of Biomedical Sciences, Faculty of Medicine, The Chinese University of Hong Kong, Sha Tin, NT, Hong Kong SAR, China

[3]Department of Assisted Reproduction, Shanghai Ninth People's Hospital, Shanghai Jiaotong University School of Medicine, Shanghai, China

[4]The International Peace Maternity and Child Health Hospital, Shanghai Jiao Tong University School of Medicine, Shanghai, China

[5]The Key Laboratory of Reproductive Genetics, Ministry of Education (Zhejiang University), Hangzhou, China

[†]These authors contributed equally to this work.

Supplementary figure legend

Supplemental Figure S1. HCO_3^- ion is required for embryo cleavage. A, Four-cell embryo formation after 12 hours of 2-cell embryo culture in TALP medium with varying concentrations of HCO_3^- and constant Cl^- concentration (115 mM) (50 mM: 27/35 embryos; 25 mM: 35/40 embryos; 12.5 mM: 15/34 embryos; 0 mM: 3/34). Scale bar: 100 μm. **B**, Summary of the results from C. ns indicates $P > 0.05$, *** indicates $P < 0.001$ (by one-way ANOVA, $n = 4$).

Supplemental Figure S2. Expression of SLC channels mRNA in 2-cell to 8-cell embryos. Quantitative real-time PCR results showing the mRNA expression level of SLC channel family members. The expression was normalized to rRNA expression in 2-cell to 8-cell embryos.

Supplemental Figure S3. Dose-dependent effects of niflumate and DIDS on inhibiting embryo cleavage. Embryos were treated with indicated concentration of niflumate or DIDS in complete TALP. a, Compared with DIDS control, $P < 0.05$-0.01; b, compared with niflumate control, $P < 0.05$-0.001; c, compared with corresponding DIDS treatment, $P < 0.05$-0.01. ($n = 4$).

Supplemental Table 1. List of primers used.

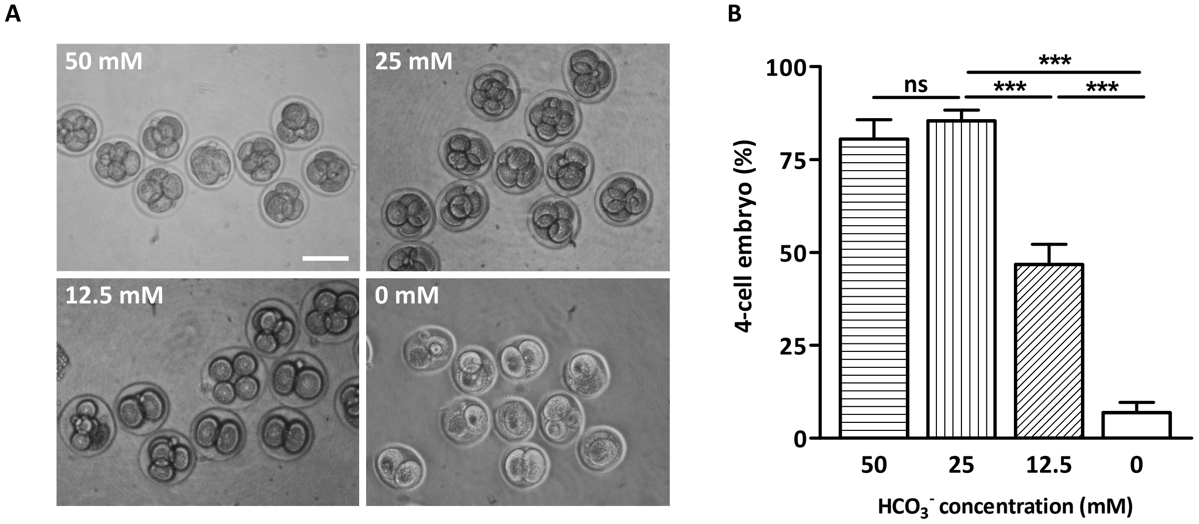

Supplemental Figure S1

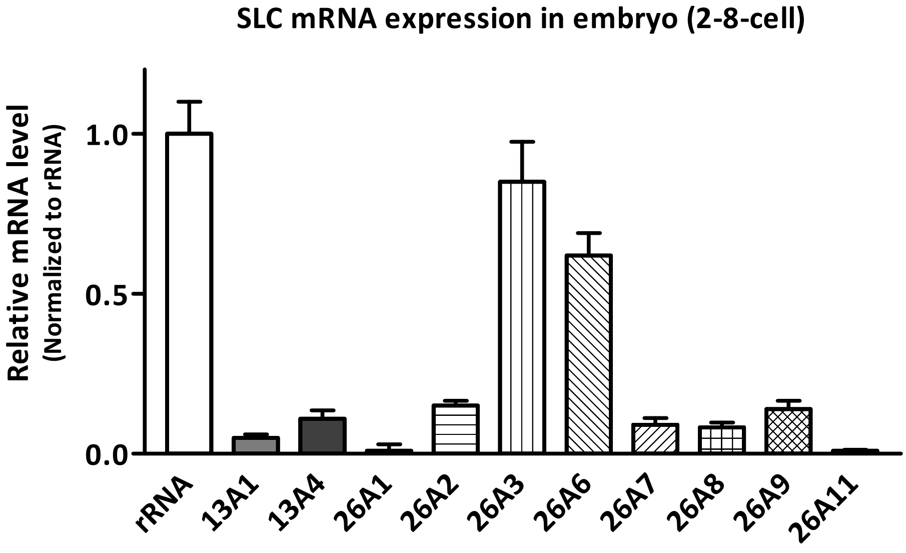

Supplemental Figure S2

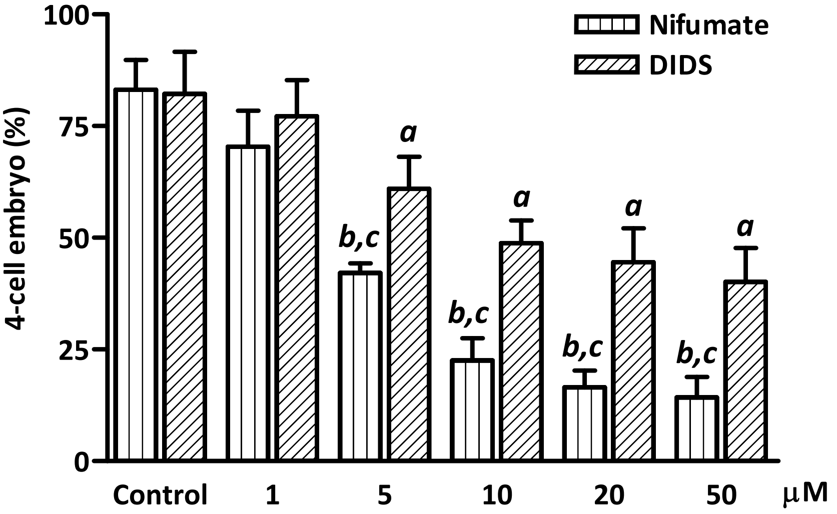

Supplemental Figure S3

Supplementary Table 1: Table Nucleotide sequences of primers used for quantitative real-time PCR (qPCR) (SYBR Green)

Target RNA	Forward (5'- 3')	Reverse (5'- 3')
Mouse SLC13A1	CTATCGCCGCTTTCTCCTTGT	GGCAGTAGAGCTGTGATTGACA
Mouse SLC13A4	GGAAGCTGCTATTGGTCATCTG	GGTCACAAGCAACACGTAAGC
Mouse SLC26A1	CAGAAGGGTGGGACACTGGTA	ACATCACCTGCTAGGTATTCCT
Mouse SLC26A2	AAGAGCAGCATGACCTCTCAC	CTGCCTCAAGTCAGTGCCT
Mouse SLC26A3	CTGAAGCGCCACACTGAATA	CTGGTGACAGTGGCTAGTGG
Mouse SLC26A6	TGCGGAGCCTTAGTTTACCG	ACTCCTCGGTAAGCAGCAAC
Mouse SLC26A7	CCCCACCGAGAAGACATTAAGC	TGAACTGCCAACATTATCCCAG
Mouse SLC26A8	TTTTGTGTACGATGTTAAGCGGA	GTGGTGCTTGAAAGTGGTGA
Mouse SLC26A9	CCCCGCTACGTGGTAGACA	AGCACCTGAAAGTGTTGCGA
Mouse SLC26A11	AGTGTATGCGGGAACACATGC	CGAAGGCGTAAGCAATCAGAG
Mouse rRNA	GTAACCCGTTGAACCCCATT	CCATCCAATCGGTAGTAGCG
Mouse p53	GTCAAGAAAGTGGGGCCTGA	TGAGTGGAATCTGGGATTGTG
Mouse p21	CCAGATCTTCCGGAGCCAGG	GAGGGGCGTCATCAAAGGAA
Mouse FGF2	ACTGTCCTGTCAGTGTTTGAT	AGTGCCACATACCAACTGGAG
Mouse GAPDH	CCCCAGCAAGGACACTGAGCAAGAG	GCCCCTCCTGTTATTATGGGGGTC
Human SLC26A3	CAGTGGCCTTTTCAGTTGCC	GGGCAGTACTCCCAGCAAAT
Human SLC26A6	TACAGTGATGCGCTGAAGCA	CCAGACATGCAGACAAAAGGG
Human GAPDH	CTTCTTTTGCGTCGCCAGCC	TCCGTTGACTCCGACCTTCA
